# The Impact of Chronic Stress Related to COVID-19 on Eating Behaviors and the Risk of Obesity in Children and Adolescents

**DOI:** 10.3390/nu16010054

**Published:** 2023-12-23

**Authors:** Iwona Piątkowska-Chmiel, Paulina Krawiec, Karolina Joanna Ziętara, Piotr Pawłowski, Marzena Samardakiewicz, Elżbieta Pac-Kożuchowska, Mariola Herbet

**Affiliations:** 1 Department of Toxicology, Faculty of Pharmacy, Medical University of Lublin, Jaczewskiego 8b Street, 20-090 Lublin, Poland; iwona.piatkowska-chmiel@umlub.pl; 2 Department of Paediatrics and Gastroenterology, Medical University of Lublin, Al. Racławickie 1 Street, 20-059 Lublin, Poland; paulina.krawiec@umlub.pl (P.K.); elzbieta.pac-kozuchowska@umlub.pl (E.P.-K.); 3 Student Scientific Association at the Department of Psychology, Faculty of Medicine, Medical University of Lublin, 20-093 Lublin, Poland; kar.zietara@gmail.com (K.J.Z.); pawlowskipiotr56@gmail.com (P.P.); 4 Department of Psychology, Psychosocial Aspects of Medicine, Medical University of Lublin, Chodźki 7 Street, 20-093 Lublin, Poland; marzena.samardakiewicz@umlub.pl

**Keywords:** chronic stress, COVID-19 pandemic, overweight, obesity, children, adolescents, eating behaviors

## Abstract

During the COVID-19 pandemic, an increase in the incidence of overweight and obesity in children was observed. It appears that unhealthy food choices, an unbalanced diet, and a sedentary lifestyle, as well as experiencing stress related to the pandemic, may be contributing to this disturbing trend. Chronic stress is a significant factor contributing to eating disorders and obesity in youngsters, involving medical, molecular, and psychological elements. Individuals under chronic stress often focus on appearance and weight, leading to negative body image and disrupted relationships with food, resulting in unhealthy eating behaviors. Chronic stress also impacts hormonal balance, reducing the satiety hormone leptin and elevating the appetite-stimulating hormone ghrelin, fostering increased hunger and uncontrolled snacking. Two systems, the hypothalamic–pituitary–adrenal axis and the sympathetic system with the adrenal medulla, are activated in response to stress, causing impaired secretion of noradrenaline and cortisol. Stress-related obesity mechanisms encompass oxidative stress, neuroinflammation, insulin resistance, and neurohormonal and neurotransmission disorders. Stress induces insulin resistance, elevating obesity risk by disrupting blood sugar regulation and fat storage. Stress also affects the gut microbiome, potentially influencing chronic inflammation and metabolic processes linked to obesity. In conclusion, chronic stress is a multifaceted risk factor for eating disorders and obesity in children, necessitating a comprehensive understanding of effective preventive and intervention strategies amid the escalating prevalence of childhood overweight and obesity.

## 1. The Impact of Chronic Stress Related to the COVID-19 Pandemic on Eating Behaviors and the Risk of Obesity in Childhood

The global consequences of the COVID-19 pandemic have profoundly affected the lives and health well-being of individuals worldwide. The extensive consequences of this unprecedented situation became visible in both the physical and psychological dimensions, giving rise to significant risks associated with non-communicable diseases. The implementation of social distancing measures during the pandemic has triggered a cascade of effects, including restrictions on outdoor physical activity, and alterations in eating habits and sleep patterns. These changes have proven to be pivotal factors contributing to the surge in cases of overweight and obesity, in adults and among children [1,2,3,4]. Growing evidence suggests that chronic stress associated with the COVID-19 pandemic has played a significant role in eating habits among the general population [1,2,3,4,5,6,7]. Stress is one of the important factors that can affect appetite food intake, eating patterns, and intestinal microbiome [8,9]. Stress-induced gut dysbiosis may influence chronic inflammation and metabolic processes that may be associated with obesity [10,11]. Furthermore, chronic stress may affect nourishment preferences toward the consumption of energy-dense foods, leading to the accumulation of abdominal adipose tissue and obesity [8]. A study involving Chinese students indicated that 24.9% of them experienced anxiety arising from the onset of COVID-19. This heightened anxiety was linked to factors such as quarantine, a lack of interpersonal contacts, the overwhelming influx of news, and exposure to false information—all contributing to an increased mental burden and a change in eating habits [12]. Clinical observations indicate that individuals dealing with chronic stress often develop a fixation on their physical appearance and weight, fostering a negative body image that disrupts their relationship with food. This discordance can give rise to maladaptive eating behaviors, such as emotional overeating—seeking solace in highly processed foods as a means of escaping stress or finding comfort. The consequences of such practices may manifest in the form of excessive calorie intake, ultimately leading to overweight and obesity. Regrettably, during the pandemic, there was a concerning rise in obesity, among children and adolescents [1]. The unintentional consequence of social distancing restrictions was a negative impact on physical activity opportunities [13,14]. A meta-analysis of 22 studies of 14,216 children aged 3–18 years indicated that during the COVID-19 pandemic, total engagement in physical activity in children was reduced by 20% (90% CI −34 to −4%) compared with the pre-pandemic period [13]. This negative phenomenon was most prominent in moderate-to-vigorous physical activity with a −28% change (90% CI: −41% to −13%) [14]. Isolation and an increase in sedentary lifestyles have played a key role in the development of excess weight among this demographic [15,16,17,18]. Moreover, it has been noticed that after the COVID-19 outbreak, screen time among children increased [19,20]. A recent report published by the CDC demonstrated that in the pediatric population in the United States between the pre-pandemic period and during the COVID-19 pandemic, the rate of body mass index almost doubled from 0.052 (95% CI = 0.051–0.052) to 0.100 (95% CI = 0.098–0.101) kg/m^2^/month [1]. It should be also emphasized that the increase in the rate of BMI change was particularly apparent in those children and adolescents who experienced overweight and obesity before the pandemic [1,6].

A meta-analysis of twelve studies from eight countries also revealed a significant association between the lockdown during the COVID-19 pandemic and gain of body weight (MD 2.67; 95% CI 2.12–3.23; *p* < 0.00001) and increase in BMI (MD 0.77 kg/m^2^; 95% CI 0.33–1.20; *p* = 0.0006) in school-age children and adolescents [2]. Based on the Children’s Hospital of Philadelphia Care Network data, it has been reported that the most significant increase in the obesity rates during the COVID-19 pandemic was recorded in children aged 5 to 9 years and those who were Hispanic, non-Hispanic Black, publicly insured, or lower income [5]. A recent systematic review revealed that although in most analyzed studies there was no reported change in the number of meals per day among children during the pandemic, there was an increase in the amount of food consumed [21]. It seems that social isolation has contributed to a rise in the consumption of home-cooked meals, linked to an increased intake of legumes, vegetables, and fruit, along with reduced consumption of fast food [18,21]. However, a noticeable surge in snacking and the consumption of “comfort food” was observed during the pandemic [18,21]. The term “comfort food” refers to a variety of meals typically considered energy-dense, high-fat, and/or high-carbohydrate products believed to provide emotional comfort [22]. Consuming comfort food during the pandemic could be seen as a coping mechanism for dealing with emotional distress, negative emotions, insecurity, and loneliness [18,21]. Furthermore, the combination of social isolation and continuous media exposure, including television even during meals, has been linked to an elevated consumption of fried food, sweets, and sugar-sweetened beverages, accompanied by a decreased intake of fruits and vegetables [18]. There is a negative relationship between screen time and physical activity and an association between extra screen time and unhealthy eating habits [23].

As indicated, chronic stress is a complex risk factor in the context of eating disorders and obesity in children and adolescents. Understanding these multidimensional aspects is crucial in developing effective preventive and intervention strategies, as the increasing prevalence of overweight and obesity in children is one of the most important challenges for health care. This review provides a comprehensive analysis of the impact of chronic stress on obesity among children and adolescents, which has significantly increased during the COVID-19 pandemic. This study takes into account both psychological and biological pathomechanisms.

## 2. Psychological Aspects of Eating Disorders during the COVID-19 Pandemic

### 2.1. Mental Health, Pandemic Environment, and Eating Disorders

Stress is an element that forms the mental sphere of every human being, regardless of age, gender, stage of mental development, or social status. It has a chronic, relatively strong, and prolonged effect on the health and biopsychospiritual status of the individual, although it can also have an individually positive effect. It such a case, stress is called eustress, which generates a positive psychological response to the interacting stressors; this effect mainly depends on personal reasoning and definition of stress and its impact on well-being [24,25,26,27].

The perception of stress by the child and adolescent is related to many factors, various social exposures, the transition between phases of psychomotor development, changes in health, family, and school situations, imposed restrictions, and peer relationships. It should be noted that, according to recent studies, a child from the age of 2 is aware of the changes occurring in their environment. Such changes include the SARS-CoV-2 (COVID-19) virus pandemic and the associated reorganization of the environment, such as isolation, homeschooling, restrictions on peer relationships, health problems for children and adolescents themselves, as well as their loved ones and others, including the topics of dying and death [28,29,30].

During the SARS-CoV-2 pandemic, the occurrence of disorders related to food intake was observed in both children and adolescents. These included problems with appetite, choice of unhealthy foods, and changing dynamics in the development of previously diagnosed eating disorders. Some of the main elements associated with abnormal relationships with food are psychological and psychiatric aspects [31,32]. The pandemic period has led to increased levels of stress, anxiety, and depression in children and adolescents, which accompany the aforementioned problems [31,32,33].

Appetite disorders include a decrease, increase, or loss of the urge to take food. A study by Paiva et al. in Brazil found a statistically significant (*p* < 0.001) association between the presence of anxiety in children (related, among other things, to social isolation during a pandemic) and the occurrence of appetite problems. The presence of anxiety in children translated into a 3.12-fold increased risk of changes in appetite, which in the majority was manifested by increased food intake [34]. A study conducted in Spain by Lavigne-Cerván et al. found that more than half of the children and adolescents studied had moderate to high levels of anxiety, which coincided with the results of a study by Jiao et al. conducted in China that showed reduced or no appetite [35,36]. The National Child Traumatic Stress Network’s guide to parents, published in 2020, highlighted the possibility of appetite-related disorders in children of all ages, indicating the nature of the problem [37]. Appetite is influenced, alongside increasing anxiety, by depressive symptoms, which are a challenge during pandemics [38,39]. Among children and adolescents suffering from depression, the majority show decreased or increased appetite, as well as weight disturbances [40,41,42].

Choosing the right food is the basis for a healthy diet. Children’s and adolescents’ dietary choices are influenced by parental eating habits, household habits, and the environment [43]. During the pandemic, access to smartphones, social isolation, and increasing stress led to the occurrence of dietary disorders. Increased intake of high-calorie foods, inter-meal snacking, and less frequent choice of healthy, low-calorie foods are caused by increasing stress and prolonged screen time [44,45,46,47].

According to the Diagnostic and Statistical Manual of Mental Disorders Fifth Edition, there are three specific eating disorders: anorexia nervosa (AN), bulimia nervosa (BN), and binge eating disorder (BED) [48]. The number of diagnoses of these disorders among children and adolescents increased during the COVID-19 pandemic, with AN being the predominant one. A cross-sectional study conducted in Canada by Agostino et al. confirmed a significant increase in new diagnoses of AN, rapid evaluation of disease markers, and hospitalizations for this condition [49,50,51]. Moreover, during the pandemic, there was also an increase in hospitalizations due to bulimia nervosa and other eating disorders [52].

It has to be highlighted that there is a mutual relationship between obesity and eating disorders, particularly bulimia nervosa and binge eating disorder [53]. Individuals with BED have a three to six times increased risk of obesity compared with the general population [54]. Although patients with BN may have normal weight, the lifetime prevalence of obesity in this group is about 33% [55]. In a cohort of Canadian adolescents, the prevalence of eating disorders was 9.3% in obese boys and 20.2% in obese girls compared with 2.1% and 8.4% of normal-weight boys and girls, respectively [56].

Obesity and eating disorders share a common pathophysiology that involves biological, environmental, behavioral, and cognitive determinants [53,57]. Possible shared genetic susceptibility for both disorders may be associated with at-mass and obesity-associated (FTO) gene polymorphisms [53]. Moreover, dysregulation of the hypothalamic–pituitary–adrenal axis (HPA) and gut dysbiosis play an important role in the pathophysiology of obesity and eating disorders [53]. Environmental risk factors identified for both disorders are, among others, weight teasing, internalization of unattainable beauty ideals portrayed on social media and television, social pressure and frequent criticism, bullying, and unhealthy family eating patterns [53,57]. Psychological determinants that may play a significant role in the development of obesity and eating disorders may include low self-esteem, negative self-evaluation, and high body dissatisfaction [53]. Body dissatisfaction, compromised interpersonal functioning, aberrant emotional regulation, and inappropriate weight control behaviors are also common factors contributing to both disorders [53,57].

Disordered eating symptomatology was exacerbatedduring the pandemic and were more pronounced in adolescents than in younger children. Individuals at increased risk for developing these conditions were also observed to exhibit their exponents. These conditions were also observed to be exhibited by individuals at an increased risk of developing them. Circumstances that may have a role in triggering eating disorders include increasing anxiety, social isolation, and more time spent on social media. This often resulted in disturbances in the perception of one’s own body; excessive physical activity, fear of gaining weight, and inappropriate food intake were often observed as a result. These circumstances not only influenced the occurrence of eating disorder symptoms but also the motivation to recover [32,58,59,60]. Family members spending more time together at home as a result of social distancing and lockdown requirements reflected increased parental recognition of eating disorder symptoms in children, which may have had an impact on the increase in acute ED visits and hospital admissions. Some patients and their families linked the onset of lockdown as a trigger for hospital admission. The overall increase in hospital admissions for eating disorder exacerbations and prolonged stays has resulted in an increased demand for care related to the treatment of these disorders [61,62,63].

Table 1 provides information on the psychological and pedagogical interventions that are used in the treatment of eating disorders in children and adolescents.

### 2.2. Prevention of Eating Disorders

In the face of the numerous described problems associated with stress-related eating disorders and obesity (covibesity) in a group of children and adolescents, preventive measures to counteract the psychosocial aspects that promote the development of these disorders become important.

**Figure 1:**
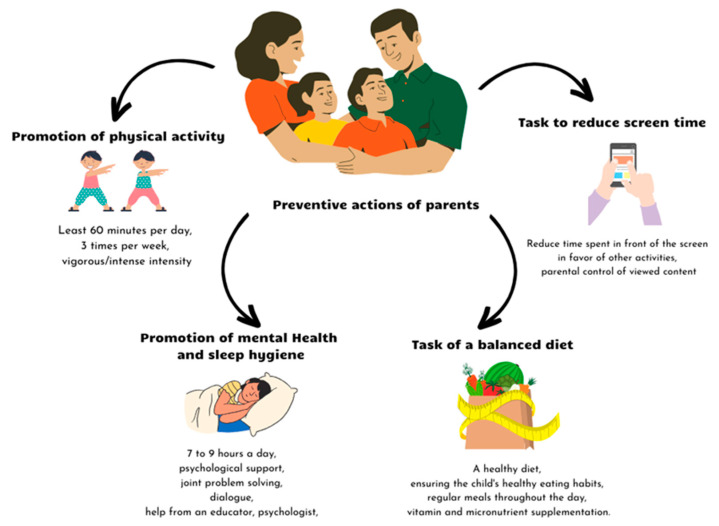
Possible covibesity prevention activities dedicated to parents [70,71,72,73,74,75,76,77,78].

The first group of recipients of the above-described measures should be the primary support system of the child or adolescent, namely the family environment. During the pandemic, adhering to the principles of social isolation, caregivers spent significantly more time at home, working remotely, supervising the learning of their offspring, and fulfilling the duties of daily life in the online system. As a result, adults became distanced from mundane pleasures and, consequently, a sense of disorganization, frustration, and anger was aroused, which was often unloaded on the child or adolescent [70,71,72]. Heightened emotional reactions from the parent, such as excessive criticism and hostility, can consequently lead to the child developing an eating disorder in the form of malnutrition or compulsive overeating [72]. Eating meals as a family can be a difficult psychological experience for a young person, and as a result, young people may restrict eating [71,72]. Available studies describe the relationship between the qualitative support of caregivers and the risk of eating disorders in their children [72]. In 2014, a program to support caregivers of people with eating disorders called Peer-Led Resilience (PiLAR) was introduced in Ireland. It was followed by an evaluation of its effects, which showed increased knowledge, skills, and improved psychological well-being of parents, positively influencing the quality of treatment and mental health of their children. Studies confirm the great importance of psychoeducation, self-help, and skills training in telepsychiatry systems in developing supportive attitudes of caregivers toward children with eating disorders and obesity [72,73]. For obesity, possible preventive actions for parents are shown in Figure 1.

Preventive actions aimed at children themselves are divided, according to the specialized literature, into two types: universal—carried out in all children regardless of the degree of assessed risk; selective—carried out in groups of high-risk children (e.g., dancers, athletes, and obese children) [74]. A meta-analysis and systematic review conducted by Chua, Tam, and Shorey confirms the effectiveness of implementing eating disorder and obesity prevention activities in school settings. These interventions are primarily aimed at reducing the internalization of beauty ideals, and thereby increasing students’ self-esteem.

These interventions are based on psychoeducation, training in the use of social media, and interpersonal training with an emphasis on the phenomena of teasing by peers. The study also indicates that girls are more prone to these activities than boys, due to their greater susceptibility to generating the cognitive illusion of a bad body image [75].

A major role in the prevention of eating disorders and obesity in the era of the COVID-19 pandemic is played by healthcare system workers, i.e., pediatricians, family physicians, and mental healthcare workers. Their roles and responsibilities are described in a narrative review by Singh et al. [76]. Pediatricians and family physicians conducting a periodic examination of a child or adolescent can recognize physical symptoms of stress and the patient’s externalizing/internalizing emotional states. These professionals can then screen for mental disorders, including eating disorders and compulsive overeating, using brief, standardized screening tools such as the Children’s Eating Disorders Inventory (EDI-C); the Eating Disorder Examination Questionnaire (EDE-Q); semi-structured interviews, such as the Children’s Eating Disorder Examination (ChEDE); and online measures, such as the Development and Well-Being Assessment (DAWBA) [77,78]. The role of mental healthcare workers is to promote mental health (in the form of digital brochures, videos, etc., available online for caregivers, teachers, and children), increase mental health awareness and, most importantly, promote the practice of mental health hygiene [38,76].

## 3. Medical Aspects of Obesity in Children and Adolescents

### 3.1. Overview of Obesity in Childhood

According to the World Health Organization (WHO), overweight and obesity are disorders characterized by abnormal or excessive fat accumulation and associated with health hazards [79]. Table 2 presents diagnostic criteria of childhood obesity according to various definitions [80,81,82,83,84,85]. Abdominal or central obesity may be identified if a waist-to-height ratio is greater than 0.5 [15,86,87]. It has been also reported that the 90th percentile of waist circumference by age and sex for children and adolescents aged 6 to 18 years is the cutoff to identify central obesity in children and adolescents [88]. Obesity is one of the most serious challenges worldwide in all age groups [15,84,87]. Joint UNICEF/WHO/World Bank estimates demonstrated that globally, in 2020, the problem of overweight and obesity referred to 5.7% of children under 5 years of age, which aligns with 38.9 million children [89].

The WHO European Regional Obesity Report 2022 indicated that in a population of children aged from 5 to 9 years, the prevalence of overweight was 29.5% and the prevalence of obesity was 11.6% [90]. Overweight occurred in 24.9% of older children and youth between 10 and 19 years of age. In this population, 7.1% of children were obese [90].

The National Health and Nutrition Examination Survey (NHANES) from 2017–2018 revealed that 16.1% of children and adolescents in the United States were overweight, and 19.3% had obesity, including approximately 30% with severe obesity [91].

Data from 2416 population-based studies in a pediatric population demonstrated that from 1975 to 2016 the increasing trend in mean BMI reached a plateau at high levels in many high-income countries, and a steep increase trend in east, south, and southeast Asia [92]. The highest prevalence of obesity exceeding 30% of the children’s population was reported in some countries of the Pacific Ocean region [92].

According to the World Obesity Federation, the global prevalence of childhood obesity is predicted to rise from 10% to 20% in boys and from 8% to 18% in girls in the period 2020–2035 [16]. It is estimated that in 2035 there will be 383 million children living with obesity worldwide [16].

Obesity has a detrimental impact on children’s health. Possible consequences of obesity in childhood are listed in Table 3 [15,84,87,93].

### 3.2. Neurochemical and Molecular Changes Induced by Chronic Stress

**Scheme 1:**
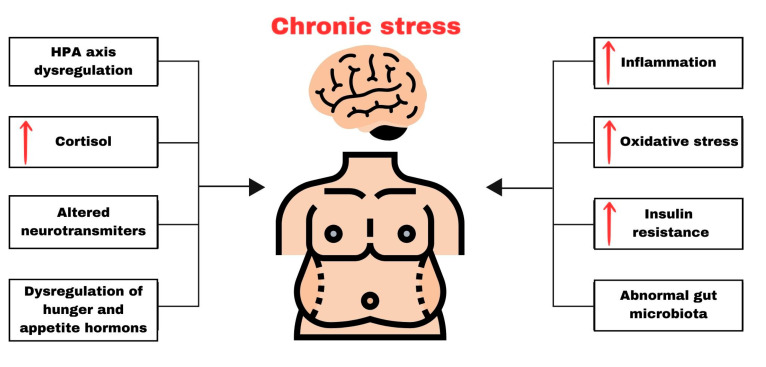
Neurochemical and molecular alterations underlying chronic stress.

Although many studies demonstrate that chronic stress is associated with obesity [94,95,96], the exact mechanisms underlying this process have not yet been fully elucidated. Molecular and neurochemical changes associated with chronic stress can indeed exert a significant influence on the development and progression of obesity (Scheme 1). This influence arises from the intricate interplay between the stress response system and a range of physiological pathways linked to the regulation of appetite, metabolism, and energy storage.

#### 3.2.1. Chronic Activation of the HPA Axis

The stress system consists of the reactions of the sympathetic nervous system, which secretes noradrenaline and adrenaline, and the hypothalamic–pituitary–adrenal (HPA) axis, which releases cortisol, playing a major role in the physiology of stress reactions [97]. The effect of adrenal corticosteroids on the secretion of adrenocorticotropin is a complex phenomenon because it depends on the type of stress. As a result of acute stress, glucocorticosteroids (GCs) directly inhibit the activity of the hypothalamic–pituitary–adrenal axis (HPA), while as a result of exposure to chronic stress, the effect of these steroids on the brain is stimulating [98]. Activation of the HPA axis activates the secretion of cortisol, a steroid hormone that regulates behavior and food choices [96]. Chronic activation of the HPA axis during chronic stress may result in prolonged cortisol effects and a subsequent orexigenic response that may manifest itself in cravings for certain types of foods [8]. In addition, GCs increase the expression of corticotropin-releasing factor (CRF) in the central nucleus of the amygdala, the emotional processing center, enabling the recruitment of the chronic stress response network. Additionally, GCs increase the perception of pleasant or compulsive activities, which motivates the consumption of “comfort foods” [98]. In this way, GCs act systemically to lead to an increase in body fat, which, in turn, leads to energy storage, inhibition of catecholamines in the brainstem, and CRF expression in hypothalamic neurons. Studies have shown that an increase in the level of the stress hormone cortisol (glucocorticoid) plays a key role in the development of obesity. Glucocorticoid redistributes white adipose tissue in a given area and increases hunger attacks and the desire to eat high-energy food, consisting mainly of fat and sugars [99]. Additionally, it has been shown that high cortisol levels can also increase appetite [100]. In turn, people with abdominal obesity experience neuroendocrine disorders, which result in impaired functioning of the hypothalamus, pituitary, and adrenal glands. In vivo studies have shown that exposure to chronic stress reduces the weight gain of animals [101]. In turn, another study showed that when rats are stressed, they are more likely to eat lard and food rich in sugar [102]. However, it has been noticed that in humans, chronic stress may have a two-way effect—it may cause increased food consumption to ensure comfort of life, which leads to weight gain. It may also suppress appetite, reduce food intake, and weight loss. Studies have shown that depressed people who consume excessive amounts of food have reduced cerebrospinal CRF, catecholamine concentrations, and hypothalamic–pituitary–adrenal activity [98]. Disturbances in the activation of the hypothalamic–pituitary–adrenal axis are related to the development of obesity because the primary basis for the increased activation of this system is hyperinsulinemia, which causes functional hypoglycemia; this, in turn, leads to increased synthesis and release of ACTH, and then to excessive adrenal production of cortisol. Adam and Epel [100] related the reward-based stress eating model, which reveals the influence of cortisol on the consumption of high-calorie food and explains the importance of potential neuroendocrine mediators in the relationship between stress and eating. Both stress and eating stimulate the release of endogenous opioids, which, in turn, leads to a decrease in the activity of the HPA axis and weakens the stress response. Repeated stimulation of the reward pathway by stress stimuli induces stimulation of the HPA axis and reaching for food. This, in turn, may lead to neurobiological adaptation of the body and compulsive eating. Cortisol, in turn, may change the level of satisfaction achieved as a result of eating through neuroendocrine mediators such as insulin, leptin, and neuropeptide Y. Physiologically, the action of glucocorticoids is counteracted by insulin and leptin; however, in chronic stress this system becomes imbalanced. This may result in increased food intake, which may lead to the accumulation of fat tissue. Therefore, it seems that excessive activation of the HPA axis may be one of the pathomechanisms linking obesity and chronic stress. Chronic activation of the HPA axis can alter glucose metabolism, promote insulin resistance, and affect many appetite-related hormones and hypothalamic neuropeptides [103]. It has been shown that people exposed to chronic stress show a greater preference for and consumption of tasty, energy-rich foods rich in sugar and fat, which contributes to the development of obesity [104].

It has also been observed that the defensive reaction caused by stress leads to increased activation of the sympathetic nervous system and vasoconstriction in skeletal muscles [105]. It has been shown that increasing sympathetic activation may contribute to the development of obesity through stress factors. Therefore, it seems that this may be another pathophysiological link between chronic stress and obesity [105]. Because food is an inexpensive and readily available source of reward that provides short-term pleasure and relief from the discomfort associated with stress responses, negative reinforcement and stress may motivate eating to regulate stress responses.

Chronic glucocorticoid exposure increases the expression and activity of lipoprotein lipase within adipose tissue depots, facilitating fat storage, and this predominates within visceral adipose tissue since visceral depots express more GR than subcutaneous depots. The stromal vascular cells of visceral adipose express higher levels of 11β-hydroxysteroid dehydrogenase (11β-HSD-1), an enzyme that regenerates glucocorticoids from their inactive metabolites, further augmenting local glucocorticoid action in abdominal fat [106]. Moreover, cortisol can increase appetite and reduce the body’s sensitivity to insulin, which leads to higher blood sugar levels [107]. Furthermore, cortisol plays a pivotal role in the activation of an enzyme called lipoprotein lipase (LPL), which results in the increased accumulation of triglycerides within fat cells, also known as adipocytes. It is essential to underscore that the level of cortisol is closely correlated with the extent of fat tissue within the body. What is of particular significance is the heightened sensitivity of intraabdominal fat tissue to the influence of cortisol, owing to the abundance of receptors for this hormone in comparison with other fat storage sites. Consequently, the adverse effects of excessive cortisol are notably pronounced in this region, thereby facilitating fat accumulation in the abdominal area [108].

Additionally, cortisol acts as an inhibitor of somatotropin, a growth hormone that typically carries out a lipolytic function under normal conditions, promoting the breakdown of fat. These intricate processes appear to be especially conspicuous in individuals afflicted by Cushing’s syndrome, characterized by elevated levels of both cortisol and insulin, along with intra-abdominal obesity. Research conducted by Rebuffé-Scrive et al. in 1988 corroborates the association between hypercortisolemia, hyperinsulinemia, and intra-abdominal obesity [109]. Similarly, Rosmond et al. (1998) validated a substantial link between postprandial salivary cortisol levels and various health parameters and obesity-related indicators in men, such as BMI, waist-to-hip ratio (WHR), fasting glucose, insulin, triglycerides, cholesterol, and blood pressure [110].

#### 3.2.2. Chronic Stress, Neurotransmitters, and Obesity

Potential mechanisms related to chronic stress and obesity include changes in the neurotransmitter system. Neurotransmitter disorder is a neurometabolic disorder that affects the way neurotransmitters are produced, broken down, or transported [111]. Neurotransmitter pathways include amino acids such as γ-aminobutyric acid (GABA), glutamate, and glycine, and monoamines such as adrenaline, noradrenaline, dopamine, and serotonin (5-HT). Chronic stress has been shown to affect brain motivation and habit-related areas [96,112]. Therefore, it is assumed that stress and reward circuits in the brain overlap and, by activating habit-based circuits, food cravings are increased by stress [96]. Catecholamine neurotransmitters such as dopamine (DA) and norepinephrine (NE) are found in brain neurons that are involved in the central nervous system’s response to stress. It has been supported that stress is associated with an increase in the activity of neurons producing both DA and NE and leads to an increase in the synthesis of both transmitters [113]. It is known that catecholamines, which are secreted by the adrenal medulla and the sympathetic nervous system, exert a significant impact on the body’s energy processes [114]. Lipolysis is initiated by catecholamines, which activate the process via β1- and β2-adrenergic receptors. This stimulation results in an elevation of intracellular cyclic adenosine monophosphate (cAMP) concentration due to cAMP-dependent activation of protein kinase [115]. HPA activation is associated with the activation of the mesolimbic dopaminergic system, a network strongly linked to the reward system. Dopamine has been linked to reward sensitivity, conditioning, and control of food abuse [103]. In people with obesity, the sensitivity of α- and β-adrenergic receptors in adipose tissue is modified. In this way, catecholamines influence the lipolytic process, leading to an increase in the storage of fat. The results of preclinical studies indicate an ambiguous response of DA to various stressful stimuli. It has been observed that acute, controlled physical stress causes increased DA outflow in the brain, while during long-term and uncontrolled exposure to the same stress factors, DA release is attenuated [116]. It is also known that chronic stress affects the neurotransmission of serotonin (5HT) in the brain. The neurotransmission of serotonin (5HT) in the brain is also known to be affected by chronic stress. Stress factors can activate raphe nuclei and rapidly increase 5HT release at efferent targets, such as the cortex, hypothalamus, and amygdala [117,118]. Therefore, chronic stress exposure may affect 5HT neurotransmission at postsynaptic targets, which may result in functional deficits in these brain regions, which, in turn, may result in abnormal behavioral patterns. Neurotransmitters such as serotonin and noradrenaline play an important role in the central nervous control of energy balance, which is why they are involved in obesity-related symptomatology. Many neurotransmitters have been shown to be involved in energy homeostasis by regulating food intake and/or energy expenditure; this phenomenon changes in the case of obesity [111]. However, whether changes in neurotransmitter metabolism are the cause or perhaps a consequence of overweight and obesity has not been fully clarified. This relationship is partially confirmed by the observation that weight gain may be caused by drugs targeting neurotransmission, such as atypical antipsychotics and antidepressants. This suggests that changes in neurotransmission may precede the onset of obesity. Another confirmation of the relationship may be the fact that the weight-loss properties of anti-obesity drugs are related to molecules acting as monoamine reuptake inhibitors or 5-HT receptor agonists and monoamine-releasing agents.

Chronic stressors have been shown to increase synaptic branching in the amygdala and anterior cingulate cortex while decreasing synaptic connections to the hippocampus and prefrontal regions [119]. Limbic regions, in turn, are involved in reward encoding and reward-based learning and feeding [120]. Repeated stressors that keep the stress system chronically activated can alter the brain’s reward pathways related to food seeking, which, in turn, can lead to metabolic changes that promote fat storage. Body weight-dependent adaptations of neural pathways may therefore enhance food preferences, appetite, and food intake under conditions of chronic stress.

#### 3.2.3. The Impact of Chronic Stress on the Neurohormonal Regulation of Appetite

The regulation of appetite is a highly complex process, finely tuned by the intricate interplay of various hormones and neurotransmitters in our body. These substances play a crucial role in governing our appetite, dictating when hunger strikes, when we reach a point of satisfaction, and even how our digestive system functions. Stress exerts a significant influence on our appetite and eating behaviors. In moments of acute stress, our appetite typically diminishes. However, the situation becomes markedly more intricate when we are under chronic stress. The prolonged periods of stress often trigger intense cravings and a pursuit of, as well as indulgence in, delicious, frequently high-calorie, and fatty foods. This phenomenon is closely intertwined with weight gain and an elevated risk of obesity.

The brain plays a central role in regulating appetite. The hypothalamus is a center that controls the feelings of hunger and satiety associated with eating. The arcuate nucleus produces various neurotransmitters such as neuropeptide Y (NPY) and agouti-related peptide (AgRP), which increase appetite, and alpha-melanocyte-stimulating hormone (α-MSH) and cocaine-and-amphetamine-regulated transcript (CART), which reduce appetite [121,122]. The paraventricular nucleus regulates the body’s energy expenditure and controls food intake based on processing information received from the arcuate nucleus. Additionally, the paraventricular nucleus produces corticotropin-releasing hormone (CRH), which affects appetite [122].

Studies have shown that neuropeptide Y participates in the regulation of, and coping with, stress and maintains emotional homeostasis, preventing the behavioral consequences of stress and anxiety. Neuropeptide Y is also one of the main neuropeptides involved in increasing appetite, the action of which is inhibited by leptin [123].

It is also worth mentioning that leptin, a hormone produced by adipocytes, exerts a significant influence on the oxidation of fatty acids. Its concentration in the bloodstream is closely linked to the amount of body fat. Upon entering the bloodstream, leptin reaches the hypothalamus, where it exerts its effects on appetite regulation. Within the hypothalamus, leptin diminishes the production of neurotransmitters that promote appetite, such as NPY/AgRP, and enhances the production of neurotransmitters that suppress appetite, like POMC/CART [124]. This leads to a reduction in food consumption and an increase in the body’s energy expenditure. Moreover, leptin not only affects the regulation of appetite and body weight but also plays an important role in maintaining the balance of blood glucose levels by increasing the sensitivity of peripheral tissues to insulin [125]. Stress is known to reduce leptin levels, a hormone released by adipose tissue that typically acts as a satiety signal to the brain. When leptin levels decrease, the body’s hunger signals can intensify, leading to excess calorie consumption [126].

Bouillon-Minois et al. (2021) showed that leptin levels decrease in response to acute stress, mainly due to its effects on the HPA axis [127]. Experiments conducted in controlled laboratory environments indicate that leptin has the capacity to suppress the secretion of corticotropin-releasing hormone (CRH). This implies that the presence of leptin in the bloodstream might act as a regulator, potentially restraining the functioning of the HPA axis [128]. It should be noted that despite higher leptin levels in people experiencing increased stress, this does not always lead to the shutdown of the HPA axis (hypothalamic–pituitary–adrenal system). The brain appears to become less sensitive to the signals transmitted by leptin. This phenomenon makes it impossible to effectively inhibit the activity of the HPA axis and control appetite [129].

Another important hormone that plays a key role in regulating appetite and influences our eating habits is ghrelin. Ghrelin is known as the “hunger hormone” and is produced mainly by the stomach lining. Typically, after we have eaten, the levels of ghrelin in our bloodstream tend to decrease, which is a signal that helps promote a feeling of fullness or satiety. However, when there is a disruption in the normal regulation of ghrelin quality by a reduced suppression of ghrelin levels (in other words, ghrelin concentrations remain elevated) after a meal, it can play a role in the tendency to overeat. This disruption in ghrelin signaling may contribute to overconsumption of food [126,130]. Recent systematic studies have shown that ghrelin concentrations increase in response to both acute mental and physical stress [127]. Moreover, it has been proven that changes in the levels of the key stress hormone, cortisol, positively correlate with ghrelin levels both before and after meals in women struggling with obesity [131]. In situations of chronic stress or when stress reaches extremely high levels, the actions of cortisol and ghrelin may work together to contribute to stressful eating habits. This includes excessive consumption of very appetizing foods and eating despite not feeling hungry.

#### 3.2.4. Chronic Stress, Inflammation, and Obesity

The relationship between chronic stress, inflammation, and obesity is complex. One of the key mechanisms through which chronic stress contributes to inflammation is the activation of the hypothalamic–pituitary–adrenal (HPA) axis. Prolonged exposure to elevated cortisol levels can disrupt the delicate balance of the immune system, resulting in an uneven distribution of pro-inflammatory and anti-inflammatory responses. This process involves immune cells, particularly macrophages, becoming active and producing pro-inflammatory cytokines like interleukin-6 (IL-6), interleukin-1β (IL-1β), and tumor necrosis factor α (TNF-α) [132]. Inflammatory processes play a disruptive role in the intricate mechanisms governing the metabolism of both fat and glucose, causing interference with the normal functioning of adipose tissue. Within the hypertrophic adipose tissue in obese individuals, nearly 50% of all cell types present are macrophages that exhibit an inflammatory phenotype. In the hypertrophic adipose tissue of obese individuals, almost half of all cell types are macrophages, and these macrophages display an inflammatory phenotype. The abundance of inflammatory macrophages within the adipose tissue significantly amplifies the ongoing state of inflammation, thereby exacerbating the intricate challenges linked to obesity. Consequently, this heightened inflammatory response often leads to a shift in fat accumulation toward visceral adipose tissue, ultimately culminating in the development of abdominal obesity, as highlighted in studies by Solinas et al. (2012) and Jager et al. (2007) [133,134]. A study by Lutz et al. (2016) showed that in people suffering from obesity, the content of macrophages in visceral adipose tissue was significantly higher compared with people with normal average body weight and amounted to as much as 12%. In people with normal average body weight, the macrophage content was only 4% [135]. Wannamethee et al. in 2007 confirmed that the concentration of IL-6 in portal veins correlates with the systemic inflammatory marker, C-reactive protein (CRP), in the blood of patients with visceral obesity [136]. Also, Illán-Gómez has confirmed a positive correlation between body mass index (BMI) and IL-6 levels in morbidly obese patients [137]. The results of a study by Arnardottir et al. (2012) confirm a strong correlation between IL-6 levels and BMI [138].

Regrettably, in the context of hypertrophied adipose tissue and the presence of pro-inflammatory mediators, the ability of adipocytes to produce not only adiponectin but also other beneficial adipokines is hampered. This, in turn, hinders the adipose tissue’s capacity to fulfill its role in regulating anti-inflammatory, anti-hyperlipidemic, and insulin-sensitizing processes [139]. Adiponectin is a substance that works in several different ways to support metabolic health. One of its important actions is to inhibit the process of lipogenesis, i.e., the creation of new lipids, by blocking the action of the sterol-binding protein-1c. Moreover, adiponectin regulates the expression of pro-inflammatory genes by inhibiting the activity of the NF-κB transcription factor. Adiponectin also promotes the activity of two important transcription factors, PPAR-α(peroxisome proliferator-activated receptor alpha) and PPAR-γ (peroxisome proliferator-activated receptor gamma). These transcription factors are responsible for increasing the process of β-oxidation (fat burning in cells) and glucose transport into cells via the type 4 glucose transporter [140]. A study by Jaleel et al. (2006) showed that obese postmenopausal women have lower levels of adiponectin compared with women with normal body weight. Additionally, the study showed that obesity was associated with metabolic abnormalities, such as disturbances in blood lipid levels and changes in leptin levels [141].

#### 3.2.5. Chronic Stress, Insulin Resistance, and Obesity

Chronic stress can negatively affect glucose homeostasis and lead to insulin resistance [142,143]. Research suggests that chronic stress may influence the development of insulin resistance in various ways. First, stress can increase the levels of hormones such as cortisol, which affect metabolism and may impair glucose regulation. Second, a notable link connection is observed between chronic stress and activation of the renin–angiotensin system (RAS) and insulin resistance. When this system is stimulated as a result of stress, there is a noticeable increase in the level of angiotensin II, a substance associated with it. Angiotensin II’s detrimental impact extends to the inhibition of insulin-stimulated tyrosine phosphorylation (IST) via a mechanism dependent on mitogen-activated protein kinase (MAPK) [144]. Additionally, angiotensin II negatively affects the availability of nitric oxide (NO), subsequently impeding insulin signaling and diminishing insulin sensitivity [145]. Furthermore, angiotensin II can diminish the activity of PI3K, a crucial component of the insulin signaling pathway [144], and it disrupts the proper localization of Glut-4 on cell membranes [144].

Another important factor is excessive stimulation of the immune system as a result of chronic stress, which leads to an increase in the level of inflammatory mediators and pro-apoptotic factors. These phenomena have the potential to damage pancreatic cells. The consequence of this is disruption of the proper functioning of pancreatic cells, which, in turn, is associated with the risk of developing metabolic disorders, including insulin resistance [146]. In a 2014 study conducted by Parkulo, it was revealed that the structure and function of pancreatic islets in mice are detrimentally affected by long-term stress, which has detrimental effects on the structure and function of pancreatic islets in mice, resulting in the shrinkage of these structures and an increased risk of type 1 diabetes. Additionally, the research showed that chronic stress diminished the activity of genes responsible for the proliferation of pancreatic beta cells [147]. Furthermore, Huffman et al. (2013) found that individuals who experienced depression and persistent mental stress exhibited impaired beta cell function, directly impacting insulin availability and glucose homeostasis [148].

#### 3.2.6. Chronic Stress, Oxidative Stress, and Obesity

In cases of chronic stress and inflammation, the equilibrium between the generation and removal of free oxygen radicals and their derivatives is disrupted, resulting in the onset of oxidative stress. Individuals enduring prolonged periods of stress often exhibit shifts in their eating habits. Stress can sometimes trigger “stress eating”, leading people to reach for high-calorie and unhealthy snacks as a way to cope with their emotional burdens. This heightened intake of fats, carbohydrates, and unhealthy fatty acids can heighten oxidative stress via diverse biochemical pathways. These processes involve the overproduction of superoxide via NADPH oxidase activity, oxidative phosphorylation, spontaneous glyceraldehyde oxidation, activation of protein kinase C, and stimulation of polyol and hexosamine pathways [149]. These metabolic abnormalities lead to increased oxidative damage to cells, which may manifest as mitochondrial and DNA damage, as well as a decrease in adenosine triphosphate (ATP) levels and lipotoxicity [150]. As a result of increased oxidative damage, there is increased production of pro-inflammatory cytokines and acceleration of lipid peroxidation. This results in increased lipogenesis and altered insulin signaling, further enhancing the production of reactive oxygen species, and creating a detrimental feedback loop [151]. Consequently, oxidative stress is a significant contributor to the excessive accumulation of body energy substances such as glucose and fats in the liver, muscles, and adipose tissue, while stimulating mitochondrial and peroxisomal oxidation processes [152]. It is worth noting that people with obesity are more susceptible to oxidative damage due to depleted resources of antioxidants, such as superoxide dismutase (SOD), glutathione peroxidase (GPx), and catalase (CAT), as well as vitamins A, E, C, and β-carotene compared with people with normal body weight [153].

Reactive oxygen species (ROS) not only stimulate the secretion of pro-inflammatory cytokines, but also activate adhesion molecules and growth factors such as connective tissue growth factor, insulin-like growth factor-1 (IGF-I), platelet-derived growth factor, and vascular cell adhesion molecule-1. This process is possible owing to the action of transcription factors that respond to changes in the level of oxidation–reduction, especially by activating the NADPH (NOX) oxidase pathway and the NF-κB factor [154]. Moreover, ROS stimulates cytokine secretion through the apurin/apyrimidin endonuclease/redox factor-1 (APE/Ref-1-)-dependent pathway [155].

It is also important to recognize that adipose tissue serves as a source of bioactive molecules known as adipokines, which can play a significant role in oxidation–reduction balance. In people who are overweight or obese, changes in the level of adipokines are often observed, especially an increase in leptin concentration. Leptin, which is secreted mainly by adipocytes, plays a role in regulating appetite and the feeling of satiety. However, in people with obesity, leptin levels are often elevated, which may lead to resistance to its effects on appetite. At the same time, increased leptin can stimulate oxidative stress by increasing phagocyte activity, inducing the synthesis of pro-inflammatory cytokines such as TNF-α, IL-6, and IL-2, and increasing the levels of endothelial cell activation markers. This may contribute to the intensification of oxidative stress in the body of people with obesity [156].

In turn, adiponectin in people with obesity reaches low concentrations in blood serum, owing to which it loses its anti-inflammatory and anti-atherosclerotic properties [157]. At the same time, adiponectin is unable to effectively limit the release of reactive oxygen species (ROS) by low-density lipoproteins (LDLs), which results in increased oxidative stress in people struggling with overweight. In individuals with obesity, inflammatory adipocytokines like visfatin and resistin are released from adipose tissue. The concentrations of these adipocytokines exhibit a positive correlation with the quantity of adipose tissue and the occurrence of oxidative stress, as evidenced by studies conducted by Moschen et al. (2007) and Chen et al. (2010) [158,159].

#### 3.2.7. Chronic Stress, Gut Dysbiosis, and Obesity

Stress is also a significant factor causing an imbalance in the gut microbiota homeostasis and gut dysbiosis [9]. The term “dysbiosis” refers to a disruption in microbiota equilibrium, leading to alterations in the composition, function, and activity of the microbial community [160]. Dysbiosis may be characterized as the loss of beneficial microorganisms, an increase in the abundance of pathobionts, and a reduction in microbial diversity [160,161]. Dysbiosis can be described as the depletion of beneficial microorganisms, a rise in the prevalence of pathogenic microorganisms, and a decrease in overall microbial diversity [160,161]. The gut microbiota plays a critical role in multiple physiological functions, including regulation of the HPA axis and sympathetic–adrenal medullary signaling, shaping the stress response [27]. Consequences of exposure to prolonged or chronic stressors in rodent models encompassed alterations in the microbiota composition and function, reduced stability of the microbiome, and increased microbial volatility [9]. Changes in the microbial community included in particular reduction in *Lactobacillaceae Akkermansia* and *Bifidobacteriacae* and an increase in pathobionts like *Clostridiacae, Escherichia,* and *Shigella* [9]. Gut dysbiosis appears to play a significant role in the development of obesity. As it has been described previously, stress is a significant factor affecting appetite and shaping dietary choices [10,11], while the diet is one of the most significant modifiable factors affecting gut microbiota homeostasis [162,163]. A high-fat diet is associated with increase in the *Firmicutes* to *Bacteriodetes* ratio, *Lactobacillus* spp., *Enterobacteriaceae*, *Bacteroidales*, *Bacteroides* spp., *Bifidobacterium* spp., and *Enterococcus* spp., and reduction in *Clostridia*, *Clostridium leptum*, and *Enterobacter* spp. in the gut microbiota composition [162]. Another implication of a high-fat diet is the low diversity of gut microbiota and the promotion of intestinal permeability [10,11]. It should be highlighted that the interaction between the diet and microbiota is bidirectional [10]. The gut microbiota with its metabolites may affect appetite regulation, reward system, or taste receptor expression [10]. Thus, it appears that stress, diet, and microbiota combine to form a virtuous cycle. Potential strategies for breaking this cycle may be focused on behavioral interventions aiming at diet modifications and stress reduction, as well as on therapeutic interventions leading to microbiota modulation.

The composition of gut microbiota in obese individuals may vary among different populations; however, in general, it is characterized by an elevated *Firmicutes* to *Bacteroidetes* ratio, and a decrease in *Bacteroides* spp., *Akkermansia* spp., and *Bifidobacterium* spp. [162,163]. Gut dysbiosis in the gut induced by a Western diet promotes obesogenic mechanisms, including the excessive harvesting extraction of energy from food and dysregulation of energy balance and gut hormones [160,163,164]. Moreover, alterations in gut microbiota may be associated with increased intestinal permeability, metabolic bacteriaemia, endotoxemia, and immune dysregulation propagating obesogenic inflammation [160,163,164]. However, further studies are needed to fully elucidate the complex role of the gut–brain axis in the pathogenesis of obesity.

## 4. Summary

The isolation policies necessary to limit the spread of SARS-CoV-2 infection have had an impact on the physical activity and eating habits of children and adolescents. Research has shown that the experience of chronic stress in the form of the COVID-19 pandemic plays an important role in many stages of the complex pathogenesis of obesity. Exposure to chronic stressors affects the neuroendocrine regulation of appetite and food preferences, and also promotes inflammation and intestinal dysbiosis, contributing to the development of obesity. It has also been shown that chronic stress influences the development of overweight and obesity through many mechanisms, such as the development of oxidative stress, neuroinflammation, insulin resistance, neurohormonal disorders, dysregulation of the HPA axis, and disorders in neurotransmission. Exposure to chronic stressors also affects the neuroendocrine regulation of appetite and food preferences and promotes inflammation and intestinal dysbiosis, contributing to the development of obesity. Obesity in children is a serious disease with serious consequences for mental, physical, and social health. It can lead to many diseases, including depression, coronary artery disease, type 2 diabetes, certain cancers, and osteoarthritis. Now more than ever, there is an urgent need to develop effective strategies to prevent overweight and obesity in the pediatric population and to develop effective multidisciplinary programs for the treatment of childhood obesity.

## Figures and Tables

**Table 1:** Psychological and pedagogical interventions used in eating disorder treatment based on [64,65,66,67,68,69].

Psychological and Pedagogical Interventions Used in Eating Disorder Treatment			
Anorexia Nervosa	Bulimia Nervosa	Binge Eating Disorder	References
Family-based treatment (FBT)	Family-based treatment (FBT)	Family-based treatment (FBT)	[64,65,66,67,68,69]
Parent-focused treatment (PFT)	Guided self-help cognitive behavioral therapy (gshCBT)	Guided self-help cognitive behavioral therapy (gshCBT)	
Systemic family therapy (SyFT)	Individual supportive psychotherapy (SPT)	Enhanced cognitive behavioral psychotherapy (CBT-E)	
Enhanced cognitive behavioral psychotherapy (CBT-E)	Cognitive behavioral therapy for adolescents (CBT-A)	Dialectical behavior therapy (DBT)	
Adolescent-focused therapy (AFT)	Psychodynamic therapy (PDT)	Interpersonal psychotherapy (IPT)	
Cognitive remediation therapy (CRT)	Dialectical behavior therapy (DBT)	Health education groups	
Intensive multifamily therapy	Interpersonal psychotherapy (IPT)		
Create a multidisciplinary team including a pediatrician, psychiatrist, psychologist, family therapist, nurse, dietician, and social services.			
Nutritional education for families.			
Psychoeducation in nutrition.			
Educational video materials.			

**Table 2:** Diagnostic criteria for childhood obesity [80,81,82,83,84,85].

Organization	Diagnostic Criteria for Obesity
World Health Organization [80,81]	Children under 5 years of age Overweight: age- and sex-adjusted weight-for-height >2 standard deviations above WHO Child Growth Standards median Obesity: age- and sex-adjusted weight-for-height >3 standard deviations above the WHO Child Growth Standards median Children between 5 and 19 years of age Overweight: age- and sex-adjusted BMI >1 standard deviation above the WHO Growth Reference median Obesity: age- and sex-adjusted BMI-for-age >2 standard deviations above the WHO Growth Reference median
U.S. Centers for Disease Control and Prevention (CDC) [82,83]	Children between 2 and 19 years of age Overweight: age- and sex-adjusted BMI ≥85th to <95th percentile on CDC Growth Charts Obesity: age- and sex-adjusted BMI ≥95th percentile on CDC Growth Charts Severe obesity: 120% of the 95th percentile of the age- and sex-adjusted BMI or greater, or BMI ≥35 kg/m^2^ Children under 2 years of age CDC recommends the use of the WHO criteria
American Academy of Pediatrics (AAP) [84]	Severe obesity Class 2 obesity: age- and sex-adjusted BMI ≥ 35 to <40 kg/m^2^ or ≥120 to 140% of the 95th percentile, whichever is lower Class 3 obesity: age- and sex-adjusted BMI ≥ 40 kg/m^2^ or ≥140% of the 95th percentile, whichever is lower
International Obesity Task Force (IOTF) [85]	Overweight International age- and sex-specific BMI percentile cutoffs equivalent to BMI 25 kg/m^2^ at age 18 years Obesity International age- and sex-specific BMI percentile cutoffs equivalent to BMI 30 kg/m^2^ at age 18 years

**Table 3:** Complications associated with obesity in childhood [15,84,87,93].

System	Disorder
Cardiovascular	Hypertension Left ventricle hypertrophy Premature atherosclerotic cardiovascular disease
Respiratory	Asthma Obstructive sleep apnea Sleep disorders Hypoventilation syndrome
Endocrine	Dyslipidemia Impaired glucose tolerance Type 2 diabetes Metabolic syndrome Polycystic ovary syndrome Impaired growth and puberty
Gastrointestinal	Metabolic dysfunction-associated steatotic liver disease Gastroesophageal reflux disease Cholelithiasis Constipation Micronutrient deficiencies
Musculoskeletal	Slipped capital femoral epiphysis Blount disease Fractures Pes planus
Skin	Acanthosis nigricans Striae Intertrigo Hidradenitis suppurativa Furunculosis
Renal	Enuresis Glomerulosclerosis
Dental	Dental caries Periodontal disease
Nervous system	Idiopathic intracranial hypertension
Psychosocial	Reduced self-esteem Depression Anxiety Disordered eating Internalizing disorders
Long-term complications	Adult obesity Coronary artery disease Type 2 diabetes Certain cancers Infertility Osteoarthritis

## Data Availability

Not applicable.
